# The mechanisms of atrial fibrillation in hyperthyroidism

**DOI:** 10.1186/1756-6614-2-4

**Published:** 2009-04-02

**Authors:** Agata Bielecka-Dabrowa, Dimitri P Mikhailidis, Jacek Rysz, Maciej Banach

**Affiliations:** 1 Department of Hypertension, Medical University of Lodz, Lodz, Poland; 2 Department of Clinical Biochemistry, Royal Free Campus, University College Medical School, University College London, London, UK; 3 Department of Nephrology, Hypertension and Family Medicine, Medical University of Lodz, Lodz, Poland

## Abstract

Atrial fibrillation (AF) is a complex condition with several possible contributing factors. The rapid and irregular heartbeat produced by AF increases the risk of blood clot formation inside the heart. These clots may eventually become dislodged, causing embolism, stroke and other disorders. AF occurs in up to 15% of patients with hyperthyroidism compared to 4% of people in the general population and is more common in men and in patients with triiodothyronine (T_3_) toxicosis. The incidence of AF increases with advancing age. Also, subclinical hyperthyroidism is a risk factor associated with a 3-fold increase in development of AF. Thyrotoxicosis exerts marked influences on electrical impulse generation (chronotropic effect) and conduction (dromotropic effect). Several potential mechanisms could be invoked for the effect of thyroid hormones on AF risk, including elevation of left atrial pressure secondary to increased left ventricular mass and impaired ventricular relaxation, ischemia resulting from increased resting heart rate, and increased atrial eopic activity. Reentry has been postulated as one of the main mechanisms leading to AF. AF is more likely if effective refractory periods are short and conduction is slow. Hyperthyroidism is associated with shortening of action potential duration which may also contribute to AF.

## Introduction

Atrial fibrillation (AF) is a common dysrrhythmia representing an independent risk factor for cardiovascular events [1]. The rapid and irregular heartbeat produced by AF increases the risk of blood clot formation inside the heart. These clots may eventually become dislodged, causing embolism, stroke and other disorders [1,2]. AF is a complex disease with several possible mechanisms. Studies indicate that arrhythmogenic foci within the thoracic veins can be AF initiators [2]. Once initiated, AF alters atrial electrical and structural properties in a way that promotes its own maintenance; this increases the risk of recurrence and may alter the response to antiarrhythmic drugs [2,3]. AF may occur in patients with a variety of cardiovascular or chronic diseases as well as in normal subjects. It is the most common cardiac complication of hyperthyroidism [3]. AF in thyrotoxicosis is associated with significant mortality and morbidity resulting from embolic events [3]. The risk factors for AF in patients with hyperthyroidism (age, male sex, ischemic heart disease, congestive heart failure and valvular heart disease) are similar to those in the general population [4]. AF occurs in up to 15% of patients with hyperthyroidism [5] compared with 4% incidence in the general population [6] and is more common in men and in patients with triiodothyronine (T_3_) toxicosis [3]. Also, subclinical hyperthyroidism is a risk factor that is associated with a 3-fold increase in risk of developing AF [5]. AF incidence increases with advancing age. Although it is rare in patients under 40 years of age, 25% to 40% of hyperthyroid individuals over the age of 60 experience AF, possibly reflecting an age-related reduction in threshold for acquiring this arrhythmia. Of hyperthyroid patients older than 60 years, 25% had AF compared with 5% prevalence in patients younger than 60 years [7]. Patients with toxic nodular goiter also showed, because of their age, an increased prevalence of AF versus younger patients with Graves' disease (43% vs 10%, respectively). Also, analysis of rhythm disorders in 219 patients with hyperthyroidism [8] showed an age-dependent distribution of AF and sinus node dysfunctions.

In a large study [9] of patients with new-onset AF, less than 1% of AF incidence was caused by overt hyperthyroidism. Therefore, although serum thyroid-stimulating hormone (TSH) is measured in all patients with new-onset AF to rule out thyroid disease, this association is uncommon in the absence of additional symptoms and signs of hyperthyroidism.

Treatment of hyperthyroidism results in conversion to sinus rhythm in up to two-thirds of patients. In a prospective trial, the arrhythmia profile was analyzed in hyperthyroid patients, before, during, and after antithyroid therapy [10]. The number of patients with atrial premature complexes was elevated compared with controls (88 vs 30%) and decreased markedly after treatment. As with other causes of AF, the main determinant of reversion seems to be the duration of AF [11]. Patients who had been in AF for more than 1 year and those who were in advanced age were likely to need intervention in the long run, probably reflecting the coexistence of ischaemic heart disease in these hyperthyroid patients with AF [12]. No association of subclinical hypothyroidism with AF has been found [5,13-15].

## The 'hypothalamic -pituitary -thyroid axis'

Thyroxine (T_4_) and triiodothyronine (T_3_) are tyrosine-based hormones produced by the thyroid gland. The major form of thyroid hormone in the blood is thyroxine (T_4_). The ratio of T_4 _to T_3 _in the blood is roughly 20 to 1. Thyroxine is converted to the active T_3 _form (3 to 4 times more potent than T_4_) by deiodinases. TSH stimulates the thyroid gland to secrete the thyroid hormones. TSH production is controlled by thyrotropin releasing hormone (TRH), which is synthesised in the hypothalamus and transported to the anterior pituitary gland via the superior hypophyseal artery, where it increases TSH production and release. Somatostatin is also produced by the hypothalamus, and has an opposite effect on the pituitary production of TSH, decreasing or inhibiting its release.

The levels of T_3 _and T_4 _in the blood have a feedback effect on the pituitary release of TSH. When the levels of T_3 _and T_4 _are low, the production of TSH is increased, and conversely, when levels of T_3 _and T_4 _are high, then TSH production is decreased. This effect creates a regulatory negative feedback loop.

## Subclinical hyperthyroidism as a risk for AF

Subclinical hyperthyroidism is defined as low serum thyrotropin concentration in an asymptomatic patient with normal serum T_3 _and T_4 _concentration [16]. The prevalence among adults is up to 12% and increases with age [16-18]. Subclinical hyperthyroidism can occur as a result of thyroid pathology such as Graves' disease, multinodular goiter, or autonomous toxic nodules or may be exogenous due to thyroxine therapy [18]. Subclinical hyperthyroidism may be a consequence of L-thyroxine (L-T_4_) therapy [19]. The abnormalities found in patients with subclinical hyperthyroidism are increased heart rate and prevalence of supraventricular arrhythmias and enhanced left ventricular mass (LVM) due to concentric remodeling [19]. The increase in LVM is associated with slightly enhanced systolic function and almost always with impaired diastolic function due to slowed myocardial relaxation [19-23]. It rarely corresponds to actual left ventricle hypertrophy and is related to the duration of subclinical hyperthyroidism rather than to circulating thyroid hormone levels. It is assumed that these changes develop in response to a chronic hemodynamic overload due to the mild hyperkinetic cardiovascular state [24].

It is reported that a low serum thyrotropin concentration in an asymptomatic person with normal serum thyroid hormone concentrations can be an independent risk factor for developing AF [24,25].

A retrospective cross-sectional study [14] compared AF prevalence in 1338 subjects with overt or subclinical hyperthyroidism due to autonomous thyroid nodules or Graves disease with AF prevalence in a control group of 22300 subjects admitted to a hospital. The prevalence of AF was 13.8% in patients with overt hyperthyroidism, 12.7% in those with subclinical hyperthyroidism, and 2.3% in euthyroid controls. The relative risk (RR) of AF in those with subclinical hyperthyroidism was 5.2 (95% CI, 2.1–8.7) compared with controls. Thus, a low serum TSH concentration is associated with a more than 5-fold higher likelihood for the presence of AF. There was no significant difference between the risk of AF in patients with subclinical and overt hyperthyroidism [14]. In another study [5] in patients older than 60 years, subjects with low thyrotropin (<0.1 mU/L) had a 28% incidence of AF compared with 11% in normal subjects. The patients with slightly lower serum thyrotropin concentration (0.1 to 0.4 mU/L) also had a higher risk of AF than those with a normal thyrotropin concentration (RR 1.6; p = 0.05). There was no significant difference in AF occurrence between overt and subclinical hyperthyroidism [5].

Similar findings have been reported by Cappola et al. [13] investigating incident AF in 3233 US community-dwelling subjects 65 years or older. After exclusion of those with preexisting AF, those with subclinical hyperthyroidism (1.6% of the cohort) had a greater incidence of AF compared with those with normal thyroid function over a period of 12 years [adjusted hazards ratio (HR), 1.98; 95% CI, 1.29–3.03]. In the study of Kwon et al. [25] the significance of serum TSH in the euthyroid patient with AF whose serum level of T_3_, T_4_, free T_4 _(fT_4_), and were absolutely within normal range was assessed. The cutoff serum TSH value that distinguished between paroxysmal and chronic AF was 1.568 U/mL (76% predictive power). There was a significantly lower serum TSH in paroxysmal AF in all age groups (p < 0.05). The authors suggest that serum TSH below the serum concentration of 1.5 U/mL can be a risk factor for developing AF.

## Overt hyperthyroidism and AF risk

In a large study including more than 23000 persons, AF was present in 513 subjects (2.3%) in the group with normal values for serum TSH, and in 78 (12.7%) and 100 (13.8%) in the groups with subclinical and overt hyperthyroidism, respectively [14].

In the study of Gammage et al. [26], serum fT_4 _was an independent predictor of the presence of AF in the cohort as a whole, and this association was sustained after exclusion of those with overt thyroid dysfunction. Furthermore, when the analysis was further restricted to those classified as euthyroid (with normal serum TSH concentration), the relationship between serum fT_4 _and AF was still evident.

In the Rotterdam Study [27] patients with high normal serum fT_4 _concentrations also had a higher risk of AF. The multivariate adjusted level of fT_4 _showed a graded association with the risk of AF (HR, 1.62; 95% CI, 0.84–3.14, highest versus lowest quartile; p for trend, 0.06).

## Effects of thyroid hormones on the cardiovascular system

Overt hyperthyroidism induces a hyperdynamic cardiovascular state (high cardiac output with low systemic vascular resistance), which is associated with a faster heart rate, enhanced left ventricular systolic and diastolic function, and increased prevalence of supraventricular tachyarrhythmias [28].

Thyroid hormones may exert both genomic and nongenomic effects on cardiac myocytes [28]. The genomic effects of thyroid hormones are mediated by transcriptional activation or repression of specific target genes that encode both structural and functional proteins [29].

Triiodothyronine (T3) is the biologically active thyroid hormone that gets into the cardiomyocyte through specific transport proteins located within the cell membrane, and which then interacts with specific transcriptional activators (nuclear receptor α-1) or repressors (nuclear receptor α-2) [30]. Occupancy of these receptors by T_3_, in combination with recruited cofactors, allows the thyroid hormone-receptor complex to bind (nuclear receptor α-1) or release (nuclear receptor α-2) specific sequences of DNA (thyroid-responsive elements) that, in turn, by acting as *cis*- or *trans*-regulators, modify the rate of transcription of specific target genes [28,31].

Severely hyperthyroid patients can show signs of congestive heart failure in the absence of prior cardiac pathology [32]. Cardiac manifestations in hyperthyroid patients can be the result of thyrotoxicosis itself, underlying heart disease that decompensates due to hyperthyroidism-induced increased demand on the heart, or increased occurrence of specific cardiac abnormalities [32]. Hyperthyroid patients frequently complain of dyspnea on exertion even in the absence of cardiac failure [33]. Because hyperthyroidism leads to a weakening of skeletal and intercostal muscles, dyspnea may be related more to a weakness of respiratory muscles than to cardiac abnormalities themselves [33-38]. Several lines of evidence suggest that some abnormalities of cardiac function in patients with thyroid dysfunction directly reflect the effects of thyroid hormones on calcium-activated ATPase and phospholamban, which are involved primarily in the regulation of systodiastolic calcium concentrations in cardiomyocytes [34]. Sarcoplasmic reticulum calcium-activated ATPase is responsible for the rate of calcium reuptake into the lumen of the sarcoplasmic reticulum during diastole that, in turn, is a major determinant of the velocity of myocardial relaxation after contraction [29,34]. It has been extensively demonstrated that thyroid hormone upregulates expression of the sarcoplasmic reticulum calcium-activated ATPase and downregulates expression of phospholamban, thereby enhancing myocardial relaxation [29,34]. The improved calcium reuptake during diastole may favorably affect myocardial contractility [34]. Some evidence indicates that thyroid hormones promote the acute phosphorylation of phospholamban and that this action attenuates the inhibitory effect of phospholamban on sarcoplasmic reticulum calcium-activated ATPase [35]. Interestingly, the fact that this process is mediated at least in part by the activation of intracellular kinase pathways involved in signal transduction of adrenaline [35] may help to explain functional analogies between the cardiovascular effects of thyroid hormone and those promoted by the adrenergic system [36]. The circadian rhythm of heart rate is maintained in thyrotoxicosis, although heart rate variability is significantly increased, supporting the view that normal adrenergic responsiveness persists in thyrotoxicosis [37].

## Electrophysiological mechanism of AF in hyperthyroidism

Several potential mechanisms could be invoked for the effect of thyroid hormones on AF risk, including elevation of left atrial pressure secondary to increased LVM and impaired ventricular relaxation [28], ischemia resulting from raised resting heart rate, and increased atrial ectopic activity [39]. Studies using an isolated heart model found that hearts from animals with experimental thyrotoxicosis show increased heart rates and shorter mean effective refractory periods than hearts from euthyroid animals [37]. In patients with hyperthyroidism increased heart rate and a decreased turbulence slope (TS) (TS quantifies the rate of sinus slowing that follows the sinus tachycardia) consistent with decreased vagal tone were observed [40].

Hyperthyroidism is associated with an increased supraventricular ectopic activity in patients with normal hearts [40]. Wustmann et al. [40] assessed the activity of abnormal supraventricular electrical depolarizations at baseline and follow-up after normalization of serum TSH levels. The abnormal premature supraventricular depolarization, the number of episodes of supraventricular tachycardia and nonsustained supraventricular tachycardia decreased significantly (p = 0.003, p < 0.0001, p = 0.01) after normalization of serum thyrotropin levels. The activation of arrhythmogenic foci by elevated thyroid hormones may be an important causal link between hyperthyroidism and AF.

Heart rate effects are mediated by T_3_-based increases in systolic depolarization and diastolic repolarization and decrease in the action potential duration and the refraction period of the atrial myocardium, as well as the atrial/ventricular nodal refraction period. T_3 _induces electrophysiological changes partly due to its effects on sodium pump density and enhancement of Na^+ ^and K^+ ^permeability [41]. Expression of the L-type calcium channel 1D, which also serves as an important pacemaker function, is also increased by T_3_.

In vitro studies found that T_3 _decreases the duration of the repolarization phase of the membrane action potential and increases the rate of the diastolic repolarization and therefore the rate of contraction [42-44].

Reentry has been postulated as one of the main mechanisms leading to AF [45-47]. Multicircuit wave fronts that are generated in the atrium could disturb normal sinus rhythm and set up a fibrillatory rhythm [45-47]. According to wavelength concepts, AF is more likely if effective refractory periods are short and conduction is slow [47]. Hyperthyroidism is associated with shortening of action potential duration [47]. Action potential duration (APD) determines the refractory period and is therefore a key determinant of the likelihood of reentry [46,47]. It has been reported that the properties of electrophysiological repolarization are not homogeneous within the 2 atria. Li et al. [48] determined that the higher density of the rapid delayed rectifier current (*I*_Kr_) in left atrial myocytes contributed to the shorter effective refractory period and APD in canine left atrium.

In several studies, changes have been observed in the expression of various ion channel mRNAs in both atria [49,50] and ventricles [49,51,52] under hyperthyroid conditions.

Watanabe et al. [50] revealed a remarkable increase in the ultrarapid delayed rectifier potassium currents in hyperthyroid compared with euthyroid myocytes, whereas the transient outward potassium currents were unchanged. L-type calcium currents were decreased in hyperthyroid compared to euthyroid myocytes. T_3 _increased the outward currents and decreased the inward currents, resulting in shortened APD.

Between the atrium and ventricle of the adult rat heart, the responses of gene expression of voltage-gated potassium channels to T_3 _were different and the variability of responses may explain cardiac manifestations of hyperthyroidism [49].

Hu et al. [53] assessed the electrophysiological changes that occur in left and right atria with hyperthyroidism, the patch-clamp technique was used to compare APD and whole cell currents in myocytes from left and right atria in both control and hyperthyroid mice. The RNAse protection assay and Western blotting were used to evaluate the mRNA and protein levels of α-subunits constituting the corresponding ion channel pore in the atrium. In hyperthyroid mice shortened APD and increased delayed rectifier currents (both the ultra-rapid delayed rectifier K^+ ^conductance ^-^*I*_Kur _and the sustained delayed rectifier K^+ ^conductance -*I*_ss_) in atrial myocytes were observed. Messenger RNA and protein expression levels of the main potential pore-forming subunits for these 2 currents, Kv1.5 and Kv2.1, were also higher in both atria in this group. It is likely that increased Kv1.5 and Kv2.1 expression reflects increased channel synthesis and at least partially contributes to the higher density of *I*_Kur _and *I*_ss _obtained under hyperthyroid conditions. The association between alteration of Kv1.5 protein but not Kv2.1 expression and mRNA level was observed in hyperthyroid atria. This suggests that thyroid hormones may regulate Kv2.1 expression at a posttranscriptional level. The influence of hyperthyroidism on APD and delayed rectifier K^+ ^currents was more prominent in right than in left atrium, which minimized the interatrial APD difference. Several factors such as differential pressure stress force, autonomic nerve innervation, or different transcriptional factor distribution between the 2 atria may play a role in this process. The overall shortening of action potential duration in hyperthyroid atria, which is reflective of a shorter effective refractory period, would facilitate the occurrence of reentry. Contrary to the normal interatrial APD difference that is important for synchronizing contraction of both atria (due to the physiological origination of sinus rhythm on the right side), the diminished interatrial APD difference may enhance the spreading of ectopic activity originating mostly from left atrium to the whole atria. Therefore, it is possible that the diminished interatrial APD would facilitate the generalization of irregular activities in a hyperthyroid state and provide the substrate for atrial arrhythmias such as AF [53]. Pulmonary veins are known to initiate paroxysmal AF [54]. Increased automaticity may also play a role in the arrhythmogenesis in hyperthyroid pulmonary veins [54]. Research using rabbit pulmonary vein cardiomyocytes has shown that thyroid hormones decrease the APD in pulmonary vein cardiomyocytes which can decrease the refractory interval and facilitate the genesis of reentrant circuits [54,55]. Incubation with thyroid hormones also increased spontaneous activity in pulmonary vein cardiomyocytes similar to its effect on sinoatrial node cells. Previous studies in humans or in isolated canine pulmonary vein tissues also have demonstrated that triggered activities may underlie the arrhythmogenic activity of pulmonary veins [55,56].

Thyroid hormones induced the occurrence of delayed after-depolarization (DAD) in beating and non-beating pulmonary vein cardiomyocytes. Transient inward currents have been suggested to play an important role in the genesis of DAD [57,58]. Tseng and Wit [57] showed that transient inward currents may play a role in the triggered activity of atrial cells in the coronary sinus. In the studies by Chen et al. [54-56] both the beating and non-beating hyperthyroid pulmonary vein cardiomyocytes had greater transient inward currents after being incubated with thyroid hormones, which may underlie the high incidence of DAD in these cells. In the beating cardiomyocytes, the incidence of early depolarization (EAD), defined as the generation of oscillatory potentials at depolarized levels, was also increased after incubation with thyroid hormones [54-56].

These findings suggest that thyroid hormones may induce the occurrence of paroxysmal AF through the increase of triggered activity in pulmonary veins. Thyroid hormones have little effects on the triggered activity of atrial cells, which suggests that these cells have different responses to thyroid hormone.

## Is it necessary to screen for thyroid function?

Regarding the high incidence of AF in older patients with thyrotoxicosis, it is important to detect thyroid dysfunction in subjects over 60 year of age. Once euthyroidism is restored, all patients who revert spontaneously to sinus rhythm (~60%) do so within 4 months of becoming euthyroid [59]. The finding that subclinical hyperthyroidism detected as a result of screening is associated with AF contributes to the debate about the value of screening at-risk populations. This debate needs to be informed by evidence from trials investigating whether treatment prevents or reverses AF [26].

Screening thyroid function tests to exclude occult hyperthyroidism as the cause of AF should include total or free T_3 _and T_4 _and high sensitivity TSH measurements.

Triiodothyronine concentration may be within the reference range in patients who are hyperthyroid and have AF [59]. The measurement of T_4 _alone can be also unsatisfactory, especially in patients with thyrotoxicosis, particularly if they have been treated for hyperthyroidism, with a nodular goitre or an autonomous thyroid nodule [60]. The TSH-producing cells of the anterior pituitary are sensitive to minor changes in circulating thyroid hormones and absent or subnormal TSH concentrations may be found in hyperthyroid patients in whom the T_3 _and T_4 _concentrations are higher than normal for the individual but within or at the upper end of the accepted reference range.

An electrocardiogram may be helpful in identifying hyperthyroid subjects at risk for developing AF. Maximum P wave duration and P wave dispersion were higher in both subclinical and overt hyperthyroidism. P maximum and P wave dispersion were significant predictors of paroxysmal AF [60-62].

## Conclusions

Several potential mechanisms could be invoked for the effect of thyroid hormones on AF risk and this association is well documented in the literature.

It is especially important to detect thyroid dysfunction in all subjects over 60 year of age, as once euthyroidism is restored all patients who revert spontaneously to sinus rhythm do so within 4 months of becoming euthyroid. The finding that subclinical hyperthyroidism detected as a result of screening is associated with AF contributes to the debate about the value of screening at-risk populations. This debate still needs to be informed by evidence from trials investigating whether treatment prevents or reverses AF [59,60].

Nevertheless in all patients with AF, before implementing pharmacological or invasive treatment, we should remember the association with thyroid diseases, as sinus rhythm is often restored after normal levels of thyroid hormones are achieved [61,62].

## Competing interests

The authors declare that they have no competing interests.

## Authors' contributions

AB-D participated in the design and the preparation of the study. DPM participated in the revisions and coordination of the study. JR participated in the design and coordination of the study. MB conceived of the study, and participated in its design, revisions and coordination. All authors read and approved the final manuscript.
